# SAXS investigation of nanoporous structure of thermal-modified carbon materials

**DOI:** 10.1186/1556-276X-9-160

**Published:** 2014-04-03

**Authors:** Bogdan K Ostafiychuk, Volodymyr I Mandzyuk, Yuriy O Kulyk, Nadiia I Nagirna

**Affiliations:** 1 Vasyl Stefanyk PreCarpathian National University, 57 Shevchenko Street, Ivano-Frankivsk 76018, Ukraine; 2 Ivan Franko National University, 8 Kyrylo and Mefodiy Street, Lviv 79005, Ukraine

**Keywords:** Porous carbon material, Small-angle X-ray scattering, Mass and surface fractals

## Abstract

The article investigates the effect of thermal modification of porous carbon material (PCM), obtained from plant feedstock, on its morphology and fractal structure by small-angle X-ray scattering (SAXS) method. The analysis of the scattering intensity curves serve the basis for calculating the parameters of the PCM porous structure: the Porod constant, the Porod invariant, average pore radius, specific surface area, and mass and surface fractal dimensions. It has been found out that the PCMs obtained have fractal structure, formed from mass and surface fractals, the sizes of which increase at the growth of temperature and modification time.

**PACS:**

81.05.Uw; 61.05.cf; 82.47.Aa

## Background

One of the principal ways to improve the existing and create new electrochemical technologies is the development of new electrode materials, possessing necessary properties: high electrocatalytic activity, stability, and abundance of original components [1]. These requirements can be provided by creating electrodes on the porous carbon material (PCM) bases that are actively used as electrode materials for primary and secondary chemical power sources and supercapacitors [2-7]. In particular, we have found out that the specific capacity of lithium power sources on the PCM bases, obtained by hydrothermal carbonization of apricot pits at different temperatures, depends mainly on its specific area and electrical conductivity [8,9]. The maximum value of specific capacity (1.138 mА · h/g) has the electrochemical system on the basis of PCM, obtained at the carbonization temperature of 750°С. It is evident that to increase the specific energy characteristic of the elements, it is necessary to perform intentional change of PCM structure and morphology by means of different types of processing and modification. The most common ways of modification are thermal, chemical, and laser modifications of PCMs [10-12]. To study changes caused by such modifications a wide range of methods are currently used: X-ray diffraction method [13], small-angle X-ray scattering (SAXS) [14-16], small-angle neutron scattering [16-18], gas adsorption/desorption [19-21], scanning tunnel microscopy [22], atomic force microscopy [23], and transmission electron microscopy [24]. Each of these methods has its advantages and disadvantages, but they provide a possibility to obtain important information about the porous structure of the materials (specific area, total pore volume, micropore volume, dimensions and forms of pores, their size distribution, fractal structure, etc.). The advantages of SAXS method, in comparison with other methods, may include the following [25,26]: (1) it is sensitive to both closed and open porosity, (2) SAXS intensity profiles are sensitive to shape and orientation of the scattering, (3) the method can be used to investigate samples that are saturated with liquids, (4) it can be used to investigate the pore texture of materials under operating conditions. Thus, the aim of the work is to perform thermal modification of PCM at different temperatures and times and to investigate the effect of this modification on its morphology and fractal structure using the SAXS method.

## Methods

The initial standard was PCM, obtained by method of hydrothermal carbonization of plant material at a temperature of 750°С. It was modified at temperatures *Т*_mod_ 300°C, 400°C, 500°C (modification time *t*_mod_ was 0.5, 1, 1.5, 2, 2.5, and 3 h), and 600°С (*t*_mod_ was 0.25, 0.5, 0.75, and 1 h) in the air in a muffle furnace SNOL-40/1300. Less PCM modification times at the temperature 600°С can be explained by the fact that at the given temperature, further thermal treatment leads to the complete material burn-off.

To determine the structural parameters of the materials investigated, the SAXS method was applied, as it is widely used to study structural heterogeneities of nanometric scope in disperse systems, including porous materials [27]. SAXS experiments were performed using X-ray diffractometer in CuK_α_ radiation (*λ* = 1.5418 Ǻ), monochromated by reflection from the (200) plane of LiF monocrystal, as X-ray beam passed through the standard. To restrict the parasitic scattering from the monocrystal monochromator and entrance slits and to reduce the intensity of the background scattering, the collimators of primary and scattered beams were used. The collimation system allows to measure SAXS spectra, starting with *s =* 0.015 Ǻ^−1^ (where $s=\frac{4\;\pi }{\lambda }\sin\theta$ and is the wave vector, and *θ* is the half of the scattering angle). The slit 0.1 mm in size was placed in front of the detector, it corresponded to the space division of the detector Δ(2*θ*)_d_ = 0.02°. The scattering radiation was recorded at the scanning mode at a step of 0.05°; the exposure interval was *τ =* 125 s. In the range of the smallest scattering angles, the scattering radiation was overlapped with the primary beam, weakened by the absorption in the standard. To exclude the influence of the primary beam on the scattering intensity, the following formula was used:

$$I^{*}\left(2\;\theta \right)=I_{\text{exp}}\left(2\;\theta \right)-T\cdot I_{\;0}\left(2\;\theta \right),$$

where I^*^(2*θ*) is the actual scattering intensity, *I*_exp_(2*θ*) is the experimental scattering intensity, *I*_0_(2*θ*) is the intensity distribution in the primary beam, and *T* = *I*_exp_(0) / *I*_0_(0) is the transmission coefficient (intensity proportion of the primary beam, passing through the standard at the zero position of detector). The obtained scattering intensity curves include the collimation adjustment for altitude of the detector receiving slit.

## Results and discussion

**Figure 1:**
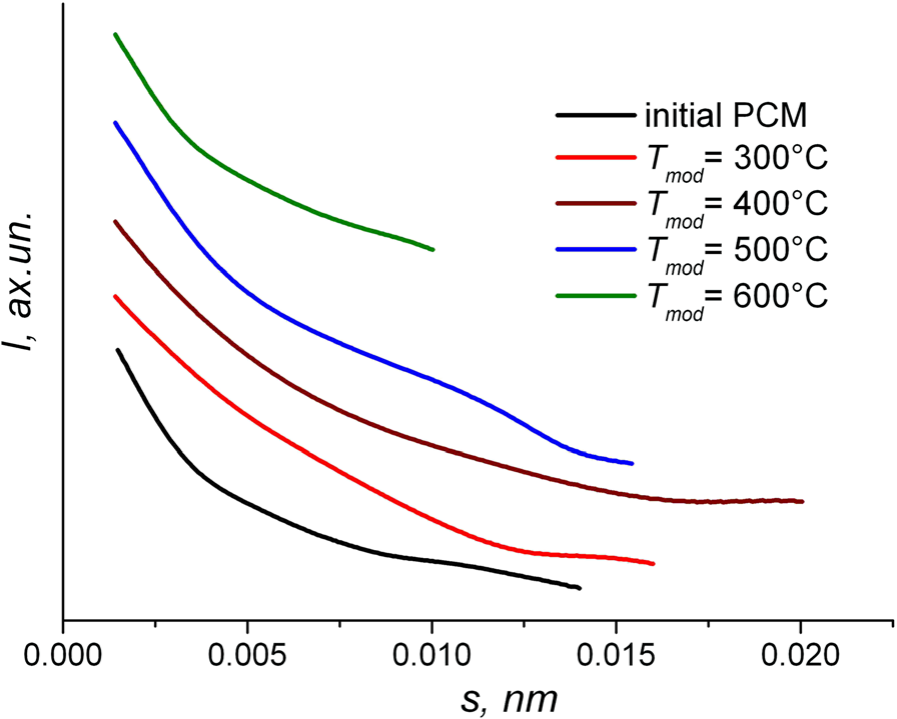
SAXS spectra of PCMs (modification time is 1 h).

As follows from the SAXS results, the obtained spectra are in the form of curves, monotonously decaying in the whole angular measurement interval. It indicates the chaotic distribution of the scattering heterogeneities (pores) and respectively the absence of correlation in their relative positions (Figure 1).

**Figure 2:**
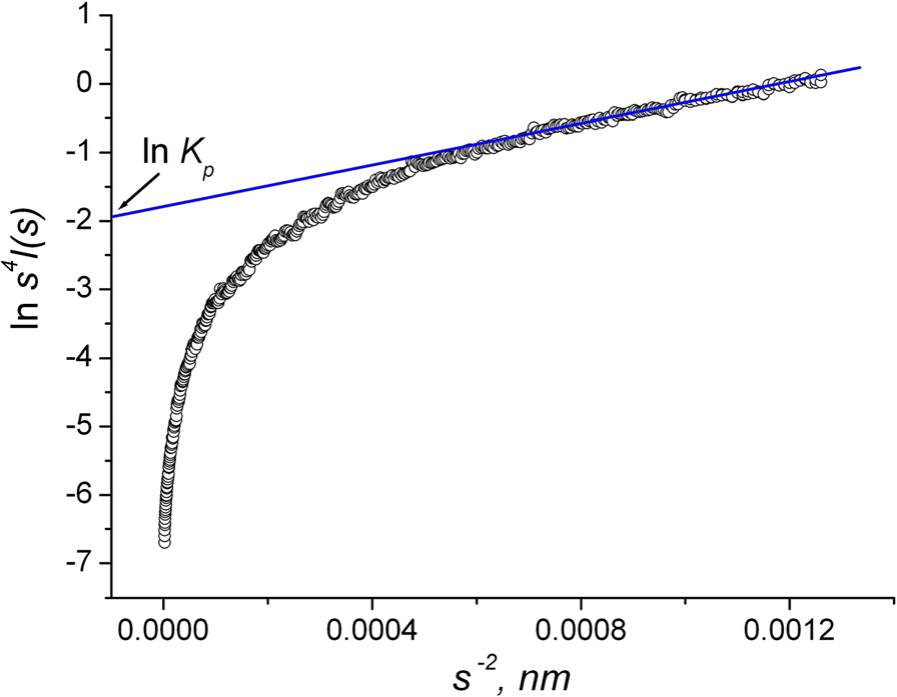
To definition of Porod constant.

To determine the parameters, characterizing the porous structure of the materials investigated, the original scattering intensity curves were analyzed. The following asymptotic Porod approximation is correct for the slit collimation system:

$$\ln\left\{s^{\;4}\;I\left(s\right)\right\}=\ln K_{\;p}+\sigma^{\;2}s^{\;2},$$

describing the behavior of the scattering intensity curves for large *s*. The parameter *σ* characterizes the state of the interphase surface. In particular, for *σ* = 0, the pore surface is smooth, for *σ* < 0 the surface is diffuse, and the value *σ* > 0 indicates the existence of fluctuations in the electron density of the material, which is characteristic of fractal objects. If there is the dependence ln {*s*^4^*I*(*s*)} vs. *s*^2^, it will described by linear function at large *s* (Figure 2). The extrapolation of this linear dependence to *s* = 0 allows us to find the Porod constant *K*_p_.

If the Porod constant *K*_p_ is known, the Porod integral invariant *Q* may be calculated by the formula

$$Q=\int_{0}^{\infty }s^{\;2}\;I\left(s\right)\;\mathrm{ds}=\int_{0}^{s_{\;0}}s^{\;2}\;I\left(s\right)\;\mathrm{ds}+K_{\;p}/s_{\;0}.$$

To calculate the effective micropore radius *R*_p_ and the specific surface area *S*_*п*_, the following formulae were used:

$$R_{p}=\frac{4Q}{\pi K_{p}},$$

$$S_{n}=\frac{\mathrm{\pi w}\left(1-w\right)}{\rho_{m}}\cdot \frac{K_{p}}{Q},$$

where *ρ*_m_ is the actual material density that in turn depends on the structural material density *ρ*_*x*_ and porosity *w* according to the equation *ρ* _m_ = (1 − *w*) *ρ* _*x*_ (structural material density is about 2 g/cm^3^).

**Figure 3:**
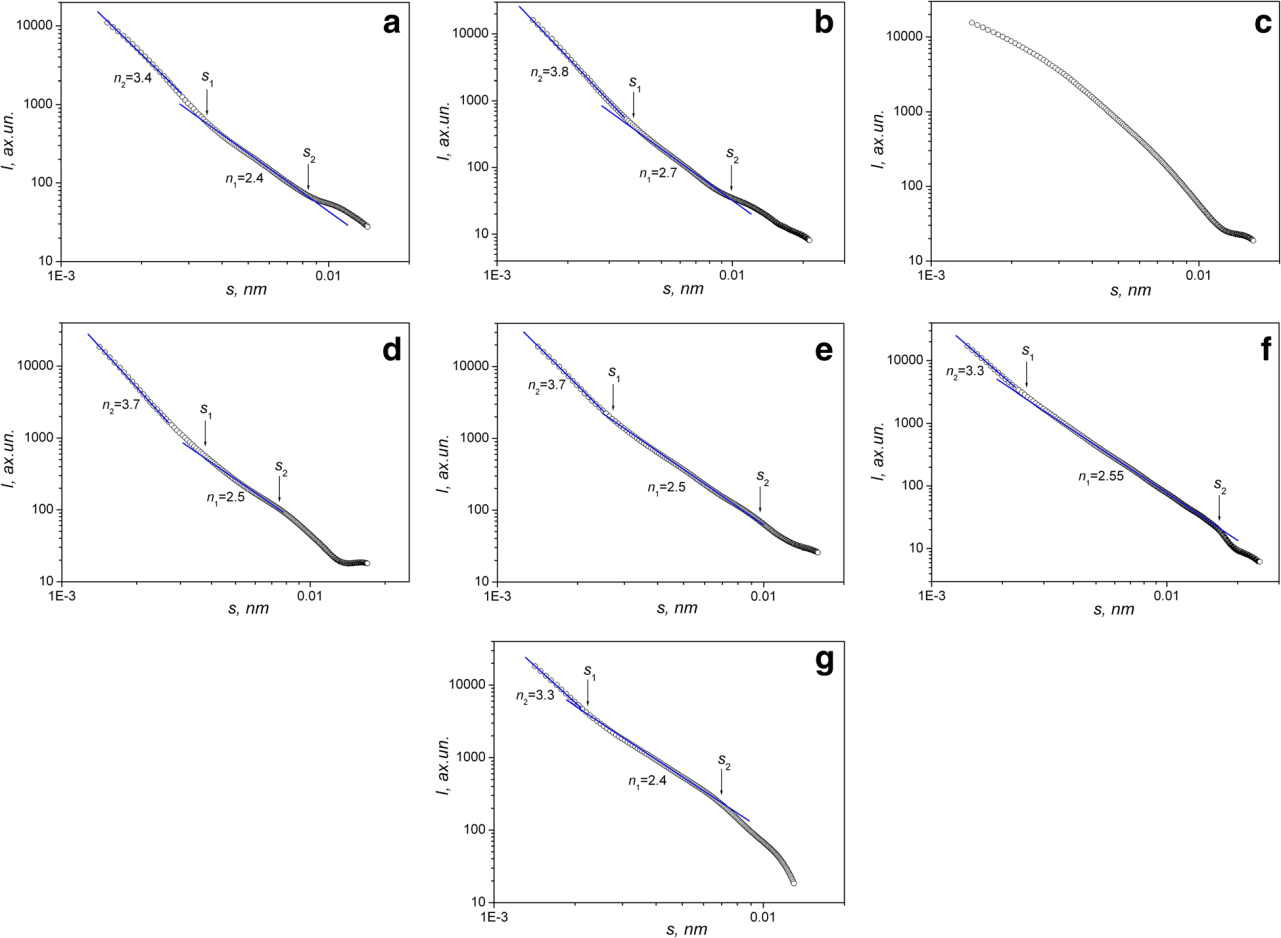
**SAXS curves in double logarithmical coordinates.** Initial PCM **(a)** and modified ones at 300°C for 0.5 h **(b)**, 1 h **(c)**, 1.5 h **(d)**, 2 h **(e)**, 2.5 h **(f)**, and 3 h **(g)**.

The results of the calculations conducted for PCM, modified at 300°С, show the non-monotonous changes in parameters of the porous structure (Table 1). The pore volume and pore surface area reach the greater value after modification for 1.5 h. In this case, the pore radius decreases up to 1.7 nm. The shape of the intensity curves of the initial standard and modified ones is similar. As can be seen from the curves in Figure 3, there are linear sections on the intensity curves the slope of which is in the range of 1 < *n* < 3. This result indicates the fractal distribution of heterogeneities. The samples contain the generated small-scale volumetric fractal structure, formed by carbon nanoclusters, the size of which can be calculated by the formula *L*_1_ ≈ 2 *π* / *s*_2_, where *s*_2_ is the lower limit of the fractal mode on the scale *s*.

**Table 1:** The parameters of porous and fractal structure of PCM modified at 300°C

***t***_**mod **_**(h)**	***Q *** **(nm**^**−3**^**)**	***K***_**p **_**(nm**^**−4**^**)**	***ρ***_**m **_**(g/сm**^**3**^**)**	***w***	***S***_***n ***_**(m**^**2**^**/g)**	***R***_**p **_**(nm)**	***L***_**1 **_**(nm)**	***L***_**2 **_**(nm)**	***D***_**v**_	***D***_**s**_
0	2,502	1,640	0.71	0.76	529	1.9	7	16	2.4	2.6
0.5	2,624	1,860	0.59	0.71	785	1.8	7	16	2.7	2.2
1	2,657	1,800	0.63	0.69	729	1.9	-	-	-	-
1.5	2,698	2,020	0.63	0.69	805	1.7	8	16	2.5	2.3
2	2,670	1,920	0.63	0.69	773	1.8	7	25	2.5	2.3
2.5	2,679	1,880	0.63	0.69	755	1.7	4	21	2.55	2.7
3	2,786	1,990	0.63	0.69	768	1.8	9	25	2.4	2.7

In addition to the small-scale structure, there forms the large-scale cluster structure, formed by clusters with the size of *L* > *L*_1_ ≈ 2 *π* / *s*_1_. The scattering from those clusters is observed in the range *s* < *s*_1_. The slope of the linear section at *s* < *s*_1_ is in the range 3 < *n* < 4, which indicates the formation of the fractal surface of large-scale carbon clusters.

As can be seen from Table 1, the fractal dimension of the cluster surface increases at the increase of material modification time. It should be noted that there are no linear section on the intensity curve of the sample modified for 1 h; that is probably the evidence of the chaotic (non-fractal) distribution of heterogeneities.

The increase of the modification temperature to 400°С and 500°С leads to the substantial change of the parameters of the porous structure of carbon materials in comparison to that modified at 300°С. The most substantial changes at the mentioned modification temperatures can be observed at the modification times 1.5 ÷ 3 h (Tables 2 and 3). First of all, it depends upon the increase of the porosity value resulting from the partial burn-off in the near-surface layers of the carbon particles and decrease of the PCMs' actual density.

**Table 2:** The parameters of porous and fractal structure of PCM modified at 400°C

***t***_**mod **_**(h)**	***Q *** **(nm**^**−3**^**)**	***K***_**p **_**(nm**^**−4**^**)**	***ρ***_**m **_**(g/сm**^**3**^**)**	***w***	***S***_***n ***_**(m**^**2**^**/g)**	***R***_**p **_**(nm)**	***R***_**с **_**(nm)**	***r***_**c **_**(nm)**	***D***_**v**_	***D***_**s**_
0	2,502	1,640	0.71	0.76	529	1.9	-	-	2.4	2.6
0.5	2,459	1,450	0.63	0.69	634	2.2	-	-	2.4	2.8
1	2,406	1,470	0.63	0.69	657	1.9	13	2.0	-	2.7
1.5	2,323	1,500	0.63	0.69	694	1.9	14	2.0	-	2.4
2	2,354	1,560	0.59	0.71	734	1.9	15	2.5	-	2.2
2.5	2,214	1,630	0.56	0.72	832	1.7	16	2.5	-	2.1
3	2,177	1,500	0.53	0.74	795	1.8	16	3.0	-	2.0

**Table 3:** The parameters of porous and fractal structure of PCM modified at 500°C

***t***_**mod **_**(h)**	***Q *** **(nm**^**−3**^**)**	***K***_**p **_**(nm**^**−4**^**)**	***ρ***_**m **_**(g/сm**^**3**^**)**	***w***	***S***_***n ***_**(m**^**2**^**/g)**	***R***_**p **_**(nm)**	***R***_**с **_**(nm)**	***r***_**c **_**(nm)**	***D***_**v**_	***D***_**s**_
0	2,502	1,640	0.71	0.76	529	1.9	-	-	2.4	2.6
0.5	2,226	1,310	0.56	0.72	665	2.2	12.5	2.5	-	2.5
1	2,237	1,500	0.53	0.74	774	1.9	14.0	3.0	-	2.4
1.5	2,273	1,510	0.53	0.74	767	1.9	14.0	2.5	-	2.2
2	2,249	1,470	0.43	0.79	806	1.9	14.0	2.0	-	2.0
2.5	2,183	1,600	0.41	0.80	915	1.7	15.0	2.0	-	2.0
3	2,230	1,610	0.39	0.81	912	1.8	15.0	1.5	-	2.0

Let us analyze the changes in the parameters of the PCM fractal structure modified at temperature 400°С (scattering intensity curves in double logarithmic coordinates for PCMs, modified at temperatures 400°С, 500°С, and 600°С, are not provided in the article, as their forms are similar to the dependences lg *I*(*s*) = *f*{lg(*s*)} in Figure 3).

The intensity curve of the sample, modified for 0.5 h, represents the linear section, the slope of which *n*_1_ = 2.4 indicates the formation of the volumetric fractal structure with the dimension of *D*_v_ = 2.4. A similar situation can be observed for the initial standard. One can assume that in the range of wave vectors (*s*_1_, *s*_2_), there is the scattering of nanoclusters, the sizes of which can be calculated by the formula *L*_0_ ≈ 2 *π* / *s*_2_ ≈ 7 nm. In the range *s* < *s*_1_, the linear section may be observed, the slope of which *n*_2_ = 2.8 indicates the formation of another system of fractal clusters with the size of *L* ≈ 2 *π* / *s*_1_ ≈ 20 nm, the distribution of which is of the volumetric character.

Thermal modification for 1 h leads to the substantial change of the fractal structure. On the intensity curve in a wide range of scattering angles, there is the linear section with the slope *n*_2_ = 3.3. Such shape of the scattering intensity is characteristic of the porous two-phase system (carbon matrix pore) with fractal interphase surface. In this case, the dimension of the fractal surface is *D*_s_ = *6* − *n*_2_ = 2.7 (Table 2). Departure from linearity at small scattering angles (*s* < *s*_1_) is caused by the transition to the Guinier mode, for which the dependence *I*(*s*) is described by the formula $I\left(s\right)=I\left(0\right)\cdot \exp\left\{-R_{\;g}^{\;2}\;s^{2}/3\right\}$, where *R*_g_ is the radius of gyration of the scattering heterogeneities. The Guinier mode corresponds to the independent scattering by carbon clusters with the radius of $R_{\;c}=\sqrt{5/3}R_{\;g}$ in the approximation of their spherical form.

In the range of *s* > *s*_2_, there is scattering of monodisperse heterogeneities with the size of *r*_c_. Similarly, the scattering at *s* > *s*_2_ is described by the Guinier formula. One can assume that the objects investigated are formed by the carbon clusters with the radius *R*_c_ and with the extended surface, which in turn, consist of nanoclusters with the radius *r*_c_. Thus, the values *r*_c_ and *R*_c_ define the lower and upper limits of the self-similarity of fractal surface. Further increase of the PCM modification time results in quantitative changes in structural parameters. In particular, the fractal dimension of the interphase surface increases, and modification for 2.5 to 3 h leads to the transition from fractal boundary to smooth one with the dimension of *D*_s_ = 2. Besides, there is the increase in the sizes of carbon nanoparticles *r*_c_ and fractal clusters *R*_c_ (Table 2).

In case of PCM, modified at 500°С, the scattering intensity curves are characterized by the linear section in the wide range of scattering angles, the slope of which changes within the limits 3 < *n*_2_ < 4. Such values *n*_2_ indicate on the scattering by the fractal surface with the dimension *D*_s_ = 6 – *n*_2_. In this case, the materials investigated can be also viewed as two-phase porous systems with the fractal interphase surface. The increase of the modification time leads to the decrease of the fractal dimension and transition to smooth interphase surface (*D*_s_ = 2) after modification for 2 h. It should be noted that the shape of the intensity curves for PCMs, modified at 400°С and 500°С, is similar. Thus, thermal modification at those temperatures leads to the formation of PCMs, formed by carbon clusters with the radius *R*_c_ and fractal surface, which in turn, consist of nanoclusters with the radius *r*_c_ (Table 3).

Thermal modification of the initial standard at 600°С, as compared to the treatment at 400°C and 500°С, leads to a more significant increase of the pore specific volume and surface area at the same modification times because of a higher heat-treatment temperature (Table 4). The analysis of the scattering intensity curves in double logarithmic coordinates shows the scattering at the interphase fractal surface with the dimension *D*_s_ = 2.55 ÷ 2.60. It is characteristic that the increase of the modification time does not change the fractal dimension of the surface. Thus, the objects investigated can be viewed also as two-phase porous structures, produced by the carbon clusters with the radius *R*_c_, formed from nanoclusters with the radius *r*_c_, and pores with the extended fractal surface.

**Table 4:** The parameters of porous and fractal structure of PCM modified at 600°C

***t***_**mod **_**(h)**	***Q *** **(nm**^**−3**^**)**	***K***_**p **_**(nm**^**−4**^**)**	***ρ***_**m **_**(g/сm**^**3**^**)**	***w***	***S***_***n ***_**(m**^**2**^**/g)**	***R***_**p **_**(nm)**	***R***_**с **_**(nm)**	***r***_**c **_**(nm)**	***D***_**v**_	***D***_**s**_
0	2,502	1,640	0.71	0.76	529	1.9	-	-	2.4	2.6
0.25	2,496	1,740	0.58	0.71	777	1.8	16	2.0	-	2.6
0.5	2,553	1,780	0.56	0.72	788	1.8	15	2.5	-	2.55
0.75	2,584	1,950	0.56	0.72	853	1.7	15	2.5	-	2.6
1	2,482	1,860	0.56	0.72	847	1.7	15	2.0	-	2.6

## Conclusions

The thermal modification of the initial material at temperature 300°С results in the formation of PCM with the fractal structure, formed by mass fractals with the dimension *D*_v_ = 2.4 ÷ 2.7, which combine in the surface fractal aggregates with the dimension *D*_s_ = 2.2 ÷ 2.7. The increase of the modification time leads to the growth in the sizes of both types of fractals.

The increase of the modification temperature to 400°С and 500°С leads to the increase of the pore volume and pore surface area. PCM, modified for 0.5 and 1 h, was formed by carbon clusters with the radius *R*_c_, which consists of the nanoclusters with the radius *r*_c_. The increase of the modification duration not only leads to the growth in the sizes of carbon nanoparticles and fractal clusters but also causes the transition from fractal to smooth boundary surface (*D*_s_ = 2) at *t*_mod_ = 2.5 to 3 h.

Thermal treatment at 600°С and less process duration leads to more substantial changes in the pore specific volume and surface area, the maximum of which is observed at *t*_*mod*_ = 0.75 h. Besides, PCM are the two-phase porous structures, produced by carbon clusters, formed from nanoclusters, and pores with the extended fractal surface. The increase of the modification duration does not change the surface fractal dimension (*D*_*s*_ = 2.55 ÷ 2.60).

## Abbreviations

PCM: porous carbon material; SAXS: small-angle X-ray scattering.

## Competing interests

The authors declare that they have no competing interests.

## Authors’ contributions

BKO performed the problem definition and participated in the discussion of the experimental results. VIM stated the choice method and subjects of investigation, participated in the analysis and interpretation of data, and wrote the paper. YOK designed and performed the SAXS experiment and calculated the parameters of PCM porous structure. NIN fabricated the initial standard and performed its thermal modification. All authors read and approved the final manuscript.

## Authors’ information

BKO is the corresponding member, a professor at the Physics and Technology Department, Vasyl Stefanyk PreCarpathian National University, Ivano-Frankivsk, Ukraine. VIM is an associate professor at the Physics and Technology Department, Vasyl Stefanyk PreCarpathian National University, Ivano-Frankivsk, Ukraine. YOK is a senior researcher at the Physics Department, Ivan Franko National University, Lviv, Ukraine. NIN is scientific researcher at the Physics and Technology, Vasyl Stefanyk PreCarpathian National University, Ivano-Frankivsk, Ukraine.
